# Mirror Movements in Amyotrophic Lateral Sclerosis: A Combined Study Using Diffusion Tensor Imaging and Transcranial Magnetic Stimulation

**DOI:** 10.3389/fneur.2020.00164

**Published:** 2020-03-06

**Authors:** Matthias Wittstock, Nora Wilde, Annette Grossmann, Elisabeth Kasper, Stefan Teipel

**Affiliations:** ^1^Department of Neurology, University Medicine Rostock, Rostock, Germany; ^2^Institute of Diagnostic and Interventional Radiology, University Medicine Rostock, Rostock, Germany; ^3^DZNE, German Centre for Neurodegenerative Diseases, Rostock, Germany

**Keywords:** amyotrophic lateral sclerosis, diffusion tensor imaging, transcranial magnetic stimulation, mirror movements, corpus callosum

## Abstract

**Objective:** Amyotrophic Lateral Sclerosis (ALS) is a neurodegenerative disorder predominantly affecting the motor system. In a number of patients, mirror movements (MMs) suggest involvement of transcallosal fiber tracts in conjunction with upper motor neuron involvement. The aim of the study was to elucidate functional and structural alterations of callosal integrity in ALS patients with MMs.

**Methods:** Nineteen patients with ALS displaying MMs and 20 controls underwent clinical assessment, transcranial magnetic stimulation (TMS), and diffusion tensor imaging (DTI). TBSS (tract based spatial statistics) was performed. We investigated ipsilateral silent period (iSP) as a measure of transcallosal inhibition, and diffusion changes in the corpus callosum and corticospinal tract (CST) as measure of structural integrity.

**Results:** In ALS patients TMS revealed a longer mean iSP latency than controls. Twelve ALS patients (63.2%) showed loss of iSP, but none of the controls. Using region of interest analysis, fractional anisotropy (FA) values of the CST were significantly lower in ALS patients compared with controls, but diffusion parameters of the corpus callosum did not differ between patients and controls. The lack of diffusion changes in the corpus callosum was confirmed in whole brain tract based statistics, assessing FA as well as mean, radial, and axial diffusivity. There was a significant negative correlation between resting motor threshold and FA values of the CST, but not between iSP and FA of the corpus callosum.

**Conclusion:** In conclusion the study failed to show microstructural changes in the corpus callosum in conjunction with MMs. One possible reason may be that functional disturbance of transcallosal pathways precede microstructural changes in the corpus callosum.

## Introduction

Amyotrophic lateral sclerosis (ALS) is a progressive neurodegenerative disease mainly characterized by a motor syndrome with variable expression of lower (LMN) and upper (UMN) motor neuron dysfunction. El Escorial criteria (1) require UMN and LMN involvement in one or more body regions for making the diagnosis of definitive ALS. UMN signs may be spasticity, enhanced or preserved tendon reflexes and extensor plantar response. Mirror movements (MMs) have repeatedly been reported in ALS (2–4), but are still not part of the diagnostic criteria. MMs are involuntary movements contralaterally to an intended finger movement. MMs can be seen in normal children up to 10 years of age, but their prevalence and intensity declines after this age (5–7) most likely reflecting maturation of the corpus callosum (8). Persistence or novel manifestation of MMs in adults can arise from a variety of etiologies. Persistent congenital MMs have been described in different conditions ranging from the absence of other neurological abnormalities to severe congenital hemiparesis in cerebral palsy (9). MMs also have been reported in a variety of other acquired conditions such as Parkinson's disease (PD) (10, 11) or stroke (12, 13). Two main hypothesizes for the development of MMs were discussed: abnormal development of the primary motor system, involving the ipsilateral corticospinal tract, and lack of contralateral motor cortex inhibitory mechanisms, mainly through the corpus callosum (9, 14). Pathophysiological basis of MMs in all these acquired clinical conditions is thought to be the result of a predominant alteration of callosal projecting pathways. Nevertheless, an additional or pre-existing cortical or pyramidal malfunctioning appears to be necessary for the development of MMs (2). Moreover, MMs have been found to be associated with reduced transcallosal inhibition (TI) as measured by transcranial magnetic stimulation (TMS) in ALS, PD, and stroke (3, 4, 15–17). Regardless of the exact pathophysiological mechanism of MMs, the most important clinical aspect is that they are reflecting CNS involvement by the underlying pathological condition. Diffusion MRI techniques, such as diffusion tensor imaging (DTI) have been established to study integrity of neuronal tracts *in vivo* in the human brain. DTI studies found alterations of fiber tract integrity in neurodegenerative diseases like Alzheimer's disease and ALS (18–21), including callosal involvement (22–24). An observational study found a consistent reduction in fractional anisotropy in the corpus callosum of ALS patients, extending rostrally, and bilaterally to the region of the primary motor cortices (23). A more recent study using DTI and TMS for characterization of neurodegeneration in ALS and concluded a complementary role as diagnostic biomarkers of UMN dysfunction (25). Ellis and colleagues found a significant increase in the mean diffusivity and reduction of fractional anisotropy along the corticospinal tract in ALS patients with correlation to disease severity and UMN involvement (26). None of these studies focused on MMs or functional connectivity in ALS.

Therefore, we studied associations of functional TMS measures with diffusion markers of structural integrity of the corpus callosum and cerebrospinal tract in a prospective sample of ALS patients displaying MMs to elucidate the pathophysiological concepts of MMs in ALS. We hypothesized that ALS patients with MMs would show impaired functional integrity of the corpus callosum, associated with decline of structural integrity markers. Such findings would support the role of callosal dysfunction and structural impairment for MMs in ALS.

## Patients and Methods

### Subjects

Nineteen patients with ALS and 20 healthy control subjects underwent clinical assessment, and MRI examinations, including DTI. Detailed demographic date and clinical characteristic of the entire study cohort are shown in Table 1. All subjects were right-handed as assessed by the Edinburgh handedness inventory (27). At the time point of examination 4 patients showed definitive ALS, 8 probable ALS and 4 possible ALS according to the revised El Escorial criteria (1). Three patients displayed lower motor neuron variant. Mean disease duration was 34.8 ± 34.8 months. Clinical assessment consisted of neurological examination with special respect of handedness; MMs were evaluated by sequential finger tapping of one hand without optic control and observation of MMs according to the procedure of Woods and Teuber (28), and the evaluation of the revised ALS functional rating scale (-R) (29). Furthermore, to address the upper motor neuron (UMN) involvement an UMN “burden” (UMNB) was calculated by totalling the number of pathological UMN signs on examination (maximum score 16) (30). For cognitive screening we used the “Montreal Cognitive assessment” (MOCA) (31). The MOCA score ranged between 18 and 29 with an average of 24.5 ± 4 for the ALS patients, and between 27 and 29 with an average of 27.6 ± 0.7 for the controls (*P* = 0.006). Controls did not have cognitive complaints and scored within 1.5 standard deviations of the MOCA age and education adjusted norm value.

**Table 1:** Demographic and clinical data of all study subjects.

	**ALS (*****N*** **=** **19)**		**Controls (*****N*** **=** **20)**		***p***
	**Mean (*SD*)**	**Range**	**Mean (*SD*)**	**Range**	
Age (years)	62.6 (10.5)	42–74	67.1 (5.4)	60–75	0.143
Gender (N male/ female)	11/8		5/5		0.714
ALSFRS-R	38.5 (4.7)		NA		
UMNB	9.4 (4.2)		NA		
MOCA	24.4 (3.9)	18–29	27.6 (0.7)	27–29	0.006
Disease duration (month)	34.8 (34.8)		NA		
El Escorial (N NA/poss/prob/def)	3/4/8/4		NA		
Phenotype (N class/UMN/LMN)	11/3/4		NA		

*ALS, amyotrophic lateral sclerosis; SD, standard deviation; ALSFRS-R, amyotrophic lateral sclerosis functional rating scale—revised; UMNB, upper motor neuron burden; MOCA, Montreal Cognitive Assessment; El Escorial, El Escorial criteria; NA, not applicable; poss, possible; prob, probable; def, definite; N, number, UMN, upper motor neuron; LMN, lower motor neuron; p, significance of Student's t-test*.

Patients and control individuals were only included in the study if written consent was given. The study was approved by the Institutional Review Board of the Medical Faculty, University of Rostock (A-2011-0026, A 2012-0083).

### Transcranial Magnetic Stimulation

We performed TMS in all patients and in 10 of 20 healthy controls. Central motor conduction time (CMCT), motor evoked potentials (MEP) amplitudes, and contralateral silent period (cSP) were determined in all ALS patients and control subjects. MEPs were recorded from the first dorsal interosseus muscle (FDI) and from the anterior tibial muscle (TA) using a standard circular coil (outside diameter 9 cm) connected to a Magstim 200 stimulator (Magstim Co., Whitland, Dyfeld, UK). For data acquisition a commercially available MEP system was employed (Brain Quick System Plus, Inomed, Erlangen, Germany). Investigation of the ipsilateral silent period (iSP) was performed with a focal coil (external loop diameter 7 cm). The coil was oriented to induce a posterior-anteriorly directed current flow to the hand area of the motor cortex; the point of optimal excitability (POE) of the FDI muscle was determined over the contralateral motor cortex; TMS was applied at the POE with 1.5 times resting motor threshold (RMT) while subjects performed a maximum tonic activation of the ipsilateral FDI muscle and while they kept the contralateral FDI muscle relaxed as published in detail previously (3). For determination of iSP latency and duration 10 trials for each hemisphere were used. After offline rectification and averaging of EMG signals, iSP parameters were analyzed.

### MRI Acquisition

MRI acquisitions of the brain were conducted using a 3-Tesla MRI scanner with a 32-channel phased-array head coil and parallel imaging capabilities (Magnetom Verio, Siemens, Erlangen, Germany, software syngo MR B17). Subjects were scanned in a single session without changing their position in the scanner. The following sequences were used: We acquired a sagittal high-resolutionT1-weighted magnetization prepared rapidly acquired gradient echo (MP-RAGE) 3D-sequence, matrix size of 256 × 256 × 192, isometric voxel size 1.0 mm^3^), TE/TI/TR of 4.82 ms/1,100 ms/2,500 ms, flip angle 7°. To identify white matter lesions a two-dimensional T2-weighted sequence was performed (fluid attenuation inversion recovery FLAIR, matrix size of 384 × 187, 24 slices with slice thickness of 5.0 mm, TE/TI/TR of 94 ms/2,500 ms/9,000 ms, flip angle 150°). Diffusion-weighted imaging was performed with an echo-planar-imaging sequence (TE/TR 81 ms/12,700 ms) Diffusion gradients were applied in 30 different spatial directions. The b values were 0 and 1,000 s/mm^2^. The images had a matrix size of 128 × 128 mm, slice thickness of 2 mm, the resulting voxel size was 2.0 × 2.0 × 2.0 mm^2^.

### MRI Data Processing

DTI data were pre-processed using the DTI tool box of the FSL software (http://www.fmrib.ox.ac.uk/fsl/, FMRIB, Oxford, UK, Version 4.1) (32). We first applied corrections for eddy currents and head motion. The skull was stripped using Brain Extraction Tool for differentiation of brain tissue and non-brain tissue using a binary mask (threshold 0.15–0.3) and the diffusion tensors were fitted to the data with DTI fit (FMRIB Image Analysis Group, Oxford, UK). Fractional anisotropy (FA), and mean diffusivity (MD) maps as well as axial diffusivity (AD) and radial diffusivity (RD) maps were created.

TBSS analysis (tract based spatial statistics) were performed using FMRIB software (version 4.1 www.fmrib.ox.ac.uk/fsl) (33). TBSS allows spatial reorientation of FA maps into a standard space without systematic effects of spatial transformation on fiber tract directionality and without the need to select a spatial smoothing kernel that may impact upon the desired effects (22). Processing of diffusivity maps consisted of the following steps: FA maps were transformed to MNI (Montreal Neurologic Institute) space. Normalized FA maps of all subjects were averaged and skeletonized with a standardized threshold of 0.2. Based on the study specific with matter skeletons an individual skeleton for each subject was created.

**Figure 1:**
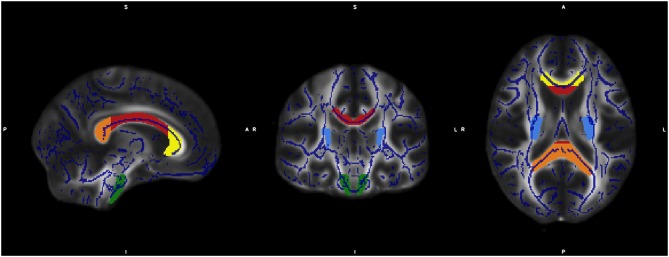
The figure shows the overlap of the mean FA skeleton map of our sample (blue) and the normalized MNI-FA template and the defined regions of interests based on JHU White matter atlas: genu (yellow), body (red), and Splenium (orange) of Corpus callosum, CST both sites (green) and crus posterior of the internal capsula on both sides (light blue).

For specific analysis of changes in diffusivity of individual fiber tracts we conducted a region of interest (ROI) analysis. The selection of ROIs from JHU was done as described before (34). Based on the JHU white matter atlas (35) we defined seven regions: corpus callosum with subregions genu, truncus, and splenium, corticospinal tract, and crus posterior of the internal capsula on both sides. Data processing was done by Matlab software 2013a (MathWorks Inc., MA, Natrick, USA) (Figure 1).

For analysis of correlation between TMS values and DTI measures we performed a region of interest (ROI) analysis using MarsBaR (36) to extract mean values from corpus callosum and left and right corticospinal tract, respectively.

### Statistical Analysis

Statistical analysis was conducted for the clinical scores, TMS measurements and ROI data in SPSS (version IBM SPSS statistics 20). For the whole brain analysis using the tool box “Randomize” under TBSS in FMRIB (version 4.1 www.fmrib.ox.ac.uk/fsl) was used. All clinical scores and TMS parameters showed a normal distribution, which was tested by the use of Kolmogorov Smirnov test (*P* > 0.1). Differences in TMS parameters were compared between groups using Student's *t*-test. The results of iSP measurements were dichotomized in pathological vs. non-pathological, and analyzed using Chi^2^ test.

For ROI analysis, group comparisons of FA of patients and controls were performed according to the findings of pathological iSP and non-pathological iSP in an univariate variance analysis and subsequent *post-hoc* tests. Furthermore, correlations between iSP findings and ALSFRS and FA parameters of all investigated ROIs was calculated. For correlation analysis Spearman-Rank-correlation were used and significance was set at *P* < 0.05.For whole brain analysis, we investigated group differences in fractional anisotropy and mean diffusivity using the general linear model on a voxel basis. Whole brain analyses were performed for all diffusion parameters. FA, MD, RD, and AD maps were compared by univariate variance analysis (factor: group, covariate: ALSFRS-R and UMNB). To avoid overestimation of significance, a test of multiple comparisons was applied (family wise error method) and significance was set at *P* < 0.05.

## Results

### TMS Results

**Figure 2:**
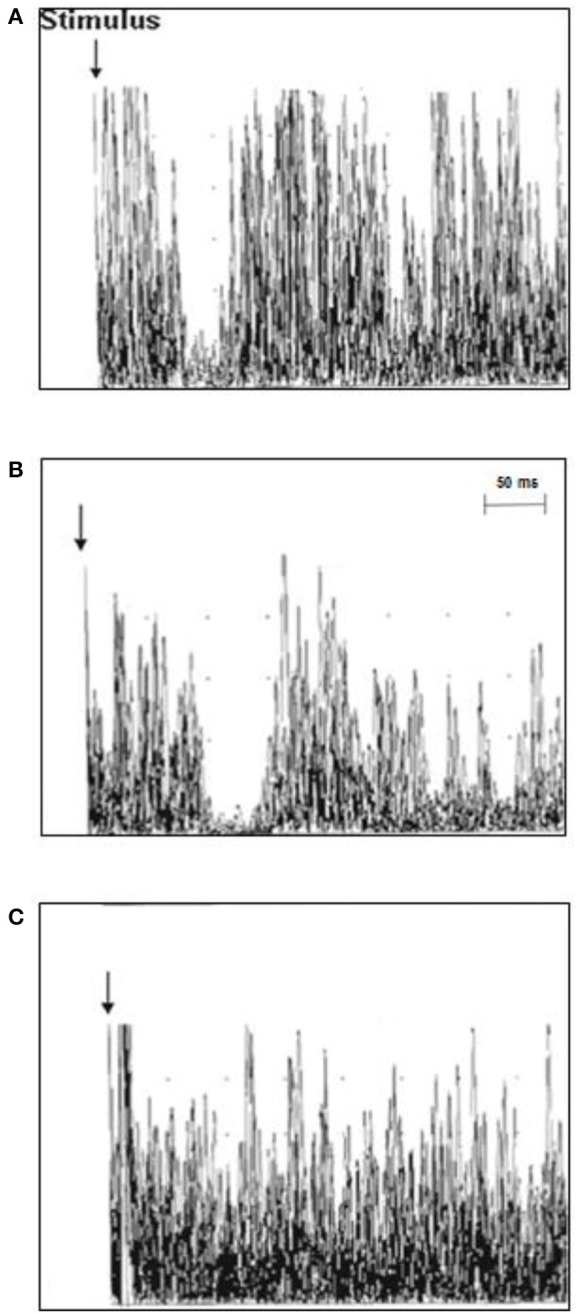
Ipsilateral muscle responses (10 rectified and superimposed EMG-traces) recorded from the first dorsal interosseous muscle (FDI) in individual ALS patients and controls. **(A)** The latency of the iSP was normal in control case 3 (37.8 ms), **(B)** the iSP latency was prolonged in ALS patient case 11 (51 ms), and **(C)** ALS patient case 18 displayed a loss of the iSP.

TMS investigation in ALS patients revealed a mean iSP latency of 41.3 ± 5.4 ms/40.9 ± 5.7 ms (right/left), which was longer than that of the healthy controls 39.0 ± 4.6/37.7 ± 6.3 (right/left) (*p* = 0.006/0.012). No differences were found for the iSP duration between the ALS patients and control subjects. Twelve ALS patients (63.2%), but none of the controls showed loss of iSP. cSP was numerically shorter in ALS patients than in controls without reaching significance. ALS patients had a significantly lower mean RMT compared to controls (TMS results are shown in detail in Table 2 and Figure 2).

**Table 2:** TMS findings in ALS patients and controls.

	**ALS (*****N*** **=** **19)**		**Controls (*****N*** **=** **10)**		***p***
	**right**	**left**	**right**	**left**	**right/left**
RMT	50.9 (16.9)	51.6 (1.3)	41.5 (4.6)	41.1 (3.5)	0.029/0.015
CMCT	8.1 (3.8)	7.9 (4.3)	6.9 (0.7)	6.8 (0.8)	0.123/0.203
iSP latency	41.3 (5.4)	40.9 (5.7)	39.0 (4.6)	37.7 (6.3)	0.006/0.012
iSP duration	18.3 (1.2)	17.2 (4.3)	12.3 (2.1)	14.3 (2.8)	0.113/0.481
iSP loss	9	8	0	0	
iSP pathological	12 (68.4%)		0		0.000
cSP	156.8 (45.1)	156.6 (41.2)	155.6 (35.3)	149.0 (28.6)	0.402/0.326

### Group Differences in Fractional Anisotropy and Mean Diffusivity

Concerning the analyses of pre-specified ROIs FA values of the CST were significantly lower in ALS patients compared with controls. FA values of the different ROIs of the corpus callosum (genu, splenium, and body) did not differ between both groups (Table 3). AD and RD values showed similar findings. Whole brain analysis did not show any differences in FA, MD, RD, and AD between ALS patients and controls at an FWE corrected level of significance.

**Table 3:** DTI findings in ALS patients and controls [fractional anisotropy (FA), mean diffusivity (MD), axial diffusivity (AD), and radial diffusivity (RD)].

	**FA values**			FA values + covariate A^*^	FA values + covariate B^*^	**MD values**		**AD values**		**RD values**	
**ROI**	**ALS** **(*N* = 19)**	**Controls** **(*N* = 20)**	***p***	***P***	***p***	**ALS (10^**−4**^)**	***p***	**ALS (x10^**−3**^)**	***p***	**ALS (x10^**−4**^)**	***p***
CST right	0.589	0.624	0.026	0.011	0.093	6.075	0.643	1.057	0.038	3.849	0.068 0.164
CST left	0.621	0.648	0.126	0.020	0.049	5.959	0.760	1.067	0.114	3.624	
Genu	0.737	0.731	0.604	0.458	0.470	7.289	0.942	1.507	0.648	3.404	0.714
Body	0.698	0.701	0.875	0.346	0.355	7.700	0.621	1.536	0.445	3.871	0.876
Splenium	0.789	0.786	0.604	0.149	0.102	7.108	0.449	1.569	0.909	2.863	0.578
Crus posterior of internal capsule right	0.700	0.716	0.484	0.972	0.188	6.509	0.066	1.285	0.085	3.338	0.165
Crus posterior of internal capsule left	0.699	0.705	0.668	0.705	0.074	6.519	0.871	2.879	0.833	3.344	0.658

* *Covariate A: ALSFRS-R, amyotrophic lateral sclerosis functional rating scale-revised; covariate B: UMNB, upper motor neuron burden*.

### Correlation of Clinical Parameter, TMS Parameters, and DTI Measures

We found significant negative correlations of FA values of the CST with RMT values on both sides in ALS patients, but not in controls (Table 4). There was no significant correlation between FA values and iSP parameters (Table 4). In contrast, there was a significant correlation of ALS-FRS-R scores with FA values of the CST on both sides (r CST-right, ALS-FRS-R = 0.58, *p* = 0.009 and r CST-left, LS-FRS-R = 0.05, *p* = 0.02), but not with FA values of the corpus callosum.

**Table 4:** Correlations between TMS and DTI.

**Correlation FA values—RMT**				
		**CST right**	**CST left**	
	***r***	−0.56	−0.52	
	***p***	0.021	0.012	
**Correlation FA values—iSP**				
**iSP latency**		**Genu CC**	**Body CC**	**Splenium CC**
**Right**	***r***	0.25	0.15	0.18
	***p***	0.26	0.51	0.41
**Left**	***r***	0.18	0.13	0.35
	**p**	0.44	0.56	0.12
**Correlation FA values—ALSFRS-R**				
		**CST right**	**CST left**	
	***r***	0.58	0.50	
	***p***	0.009	0.02	

## Discussion

Lower and upper motor neuron dysfunction is a clinical hallmark of ALS. Besides involvement of primary motor areas, an early callosal dysfunction has been suggested in ALS as well (37). MMs may be a clinical sign of dysfunctional transcallosal pathways and could be observed in ~30% of ALS patients. Further evidence for the interpretation of MMs as markers of callosal dysfunction comes from a range of TMS studies showing callosal dysfunction as indicated by impaired transcallosal inhibition (2–4, 15) in conjunction with the presence of MMs in ALS and other conditions like Parkinson's disease (16). Furthermore, several DTI studies have shown microstructural changes not only in the pyramidal tract, but also in the corpus callosum in ALS (22, 23, 38). Callosal involvement has been demonstrated to be a relatively consistent feature of ALS, even without clinical UMN involvement, and may reflect interhemispheric spread of pathology and an impaired structural motor connectivity (23, 24).

In the current study we expected a significant correlation of DTI measures and TMS findings reflecting transcallosal inhibition in ALS patients with MMs. Consistent with our expectation, we found an impaired transcallosal inhibition in up to 68% of cases which is in line with previously published data from independent cohorts (3, 4). In contrast, however, ALS patients with MMs in the current sample did not show microstructural involvement of the corpus callosum, which is in contrast with some (22, 34), but not all previous studies (39). One interpretation of the lack of corpus callosum changes in our study may be that our patients exhibited an earlier functional disturbance potentially preceding the microstructural findings in transcallosal pathways. Thus, the two previous studies finding corpus callous diffusion changes (23, 41) included functionally more advanced cases (mean ALSFRS-R score 33.1 and 36.5, respectively, compared with 38.5 in the current study). A recent study demonstrated increased AD in the corpus callosum and reduction of FA in the right CST in ALS without considering MMs (25). The study of Geraldo et al. had no focus on electrophysiological changes in the transcallosally projecting pathways but cortical excitability and CST conduction properties were tested by investigation of the resting motor threshold (RMT) as measure of cortical excitability and the central motor conduction time (CMCT). Keeping in mind that Geraldo and co-workers used a different small hand muscle as target muscle (abductor digiti minimi vs. first dorsal interosseus muscle in our study) RMT was slightly lower in our study reflecting a greater cortical excitability in our cohort displaying MMs. CMCT measurements displayed comparable values to our study. Extending these previous findings to ALS patients with MMs, we could replicate FA reduction of the CST associated with TMS changes in ALS, without effects in the corpus callosum. Another explanation of the lack of significance of DTI findings in the corpus callosum in our sample might be the relative great heterogeneity of patients, which represents one limitation of our study. Another limitation is the low number of patients. A major reason for this circumstance might be the relative rarity of MMs, which could be observed in just approximately 30% of ALS patients. Because of limitations in available staff resource the study period could not be extended.

In conclusion, this is the first study investigating a cohort of ALS patients which all were displaying MMs. The present study replicates and extends previous findings on reduced fiber tract integrity in patients with ALS with MMs, but failed to show microstructural changes accompanying mirror movements and disturbed transcallosal inhibition. The affection of the pyramidal tract appears to be an important precondition for development of MMs. This study adds further evidence for the understanding of impaired connectivity in ALS patients. Therefore, further studies in bigger and more homogeneous cohorts are needed.

## Data Availability Statement

Deidentified participant data will be shared, as well as the study protocol and statistical analyses, upon reasonable requests.

## Ethics Statement

The studies involving human participant were reviewed and approved by the Institutional Review Board of the Medical Faculty, University of Rostock (A-2011-0026, A267 2012-0083). The patients/participants provided their written consent to participate in this study.

## Author Contributions

MW: conception, design, drafting of manuscript, acquisition of data, and final approval of manuscript. NW, AG, and EK: acquisition of data, revision of manuscript, and final approval of manuscript. ST: concept, design, analysis, and interpretation of data, drafting of manuscript, revision of manuscript, and final approval of manuscript.

### Conflict of Interest

ST has done the listed works below (all in Germany): MSD Sharp & Dohme GmbH, Lindenplatz 1, 85540 Haar

11.09.2018—Quality circle for physicians in Kühlungsborn, Talk: “Dementia and Diabetes—current report”

14.11.2018—MSD Expert-forum: NAB Alzheimer in Munich, participator as consultant

13.08.2019—Event “Diabetes and Dementia” in Rostock, Talk: “Dementia and Diabetes—current report” ROCHE Pharma AG, Emil-Barell-Str. 1, 79639 Grenzach-Wyhlen

12.09.2019-−3. National Advisory-Board in Frankfurt am Main, participation as consultant

27.09.2019—ROCHE Symposium at the DGN Congress in Stuttgart, Talk: “Amyloid as target for diagnosis and treatment in Alzheimer's disease.” The remaining authors declare that the research was conducted in the absence of any commercial or financial relationships that could be construed as a potential conflict of interest.
